# Soft and wrinkled carbon membranes derived from petals for flexible supercapacitors

**DOI:** 10.1038/srep45378

**Published:** 2017-03-31

**Authors:** Xiuxiu Yu, Ying Wang, Li Li, Hongbian Li, Yuanyuan Shang

**Affiliations:** 1 School of Physical Engineering, Zhengzhou University, Zhengzhou, Henan, 450052, China; 2 CAS Center for Excellence in Nanoscience, 11, Beiyitiao, Zhonguancun, Beijing, 100190, China

## Abstract

Biomass materials are promising precursors for the production of carbonaceous materials due to their abundance, low cost and renewability. Here, a freestanding wrinkled carbon membrane (WCM) electrode material for flexible supercapacitors (SCs) was obtained from flower petal. The carbon membrane was fabricated by a simple thermal pyrolysis process and further activated by heating the sample in air. As a binder and current collector-free electrode, the activated wrinkled carbon membrane (AWCM) exhibited a high specific capacitance of 332.7 F/g and excellent cycling performance with 92.3% capacitance retention over 10000 cycles. Moreover, a flexible all-solid supercapacitor with AWCM electrode was fabricated and showed a maximum specific capacitance of 154 F/g and great bending stability. The development of this flower petal based carbon membrane provides a promising cost-effective and environmental benign electrode material for flexible energy storage.

To meet the ever-growing demand of clean energy, energy storage is highly desirable. Supercapacitors (SCs) with high power density, fast charging rate and excellent cycling performance, have attracted great attention as a high-efficient energy storage device^1^,^2^,^3^. In SCs, electrical double layer capacitor (EDLC) is one of the main type, which mainly bases on the accumulation of ions at the interface between electrode and electrolyte^4^. For the electrode materials of EDLC, porous carbon has been considered as an ideal candidate due to its high specific surface area (SSA), good conductivity and electrochemical stability. Particularly, in recent few years, nanocarbons like carbon nanotubes and graphene have been extensively explored as the electrode materials for EDLCs with excellent electrochemical performance^5^,^6^,^7^,^8^,^9^. However, the synthesis of these nanocarbons suffers from the drawbacks of the high cost chemical vapor deposition (CVD) process or the environmental issues related to the usage of toxic chemicals. Therefore, development of green methods to fabricate low-cost and high performance carbon materials in large scale is critical for the practical application of EDLCs.

Biomass derived carbon provides a promising electrode material with a low cost and environmental friendly fabrication process^10^. A range of biomasses, such as leaves^11^, pomelo peels^12^, willow catkins^13^,^14^, chicken eggshell membranes^15^, seaweeds^16^, elm samara^17^, silk^18^,^19^, sugar cane bagasses^20^ and coffee beans^21^ have been used as the precursor to fabricate porous carbon with high specific capacitance and cycling stability. However, polymer binders are always used to be mixed with these porous carbon materials to form the electrodes, which requires extra steps and also obstructs pores, resulting a reduced specific capacitance^22^. To avoid the extra mixing process and simplify the device fabrication process, a freestanding, binder and current collector-free carbon electrode is highly attractive.

Recently, freestanding carbon electrodes have been fabricated from carbonization of bacterial cellulose^23^, watermelon^24^ and cotton^25^. However, these carbon electrodes are in the form of a three-dimensional porousblock, showing limited flexibility in the as-prepared devices. Flower petals, as one of the most abundant biomass sources, can be carbonized into a freestanding carbon membrane by a simple one-step thermal pyrolysis process. Unlike other flat nature materials, the thickness of the flower petals is only 10–20 μm, resulting in a high flexibility of the carbonized petal membranes. Another advantage of these carbon membranes is that they inherit the wrinkled surface of the flower petals, which show high SSA for ions storage. Therefore, flower petal based carbon membranes are very promising as the electrodes for flexible SCs.

In the present work, flexible and wrinkled carbon membrane (WCMs) were membranes prepared by carbonizing flower petals for the first time. After an activation process by heating the WCMs in air, AWCMs with a high specific surface area (509 m^2^/g) were obtained. Unlike carbonaceous materials derived from other reported biomasses, no additional binder and current collector are needed, which greatly simplified the device fabrication process of the supercapacitors. The AWCM exhibits a maximum specific capacitance of 332.7 F/g at 10 mV/s and excellent cycling performance. Furthermore, the carbon membrane is highly flexible and a symmetric all-solid SC with high flexibility was fabricated, which exihibits a specific capacity of 154 F/g, outperforming other porous carbon based SCs. The performance of the device under bending is very stable and no apparent capacitance decay was found after 10 bending cycles.

## Results and Discussion

### Synthesis and morphologies of wrinkled carbon membranes. 

**Figure 1:**
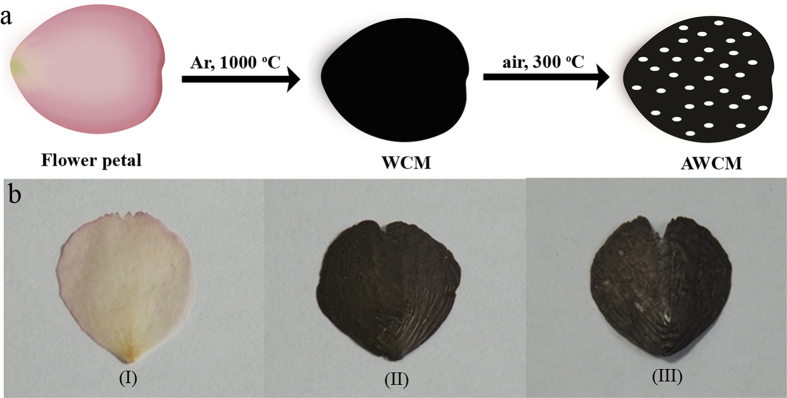
(**a**) Schematic illustration of the fabrication process of the WCM and AWCM, where a carbonization and activation two-step method was adopted. (**b**) The images of a petal at different stages: (I) dry petal, (II) carbonized petal and (III) activated carbonized petal.

The flower petals used in this paper are those from Cherry blossom collected in the campus of Zhengzhou University. Preparation of WCM and ACWM is briefly illustrated in Fig. 1a and the detailed experimental description is provided in the methods part. WCM was prepared by a thermal pyrolysis process at 1000 °C under the protection of Ar. After the carbonization process, the color of the petal changed from light pink to black, indicating the successful transformation of the petal into carbon membrane. To increase the SSA of the WCM, the as prepared carbon membrane was further heated at 300 °C in air. The activation mechanism is mainly based on the removal of active carbon atoms at the defects, which is similar as the pore fabrication process in graphene^9^. As shown in Fig. 1b, both WCM and AWCM maintained well the original structure of the petal, indicating a good mechanical strength as a freestanding membrane for EDLCs. Compared with those carbon materials activated by KOH and ZnCl_2_ that only carbon powder can be obtained^15^,^20^, mildly thermal treatment of the petals in air is beneficial to maintain their unique structure.

**Figure 2:**
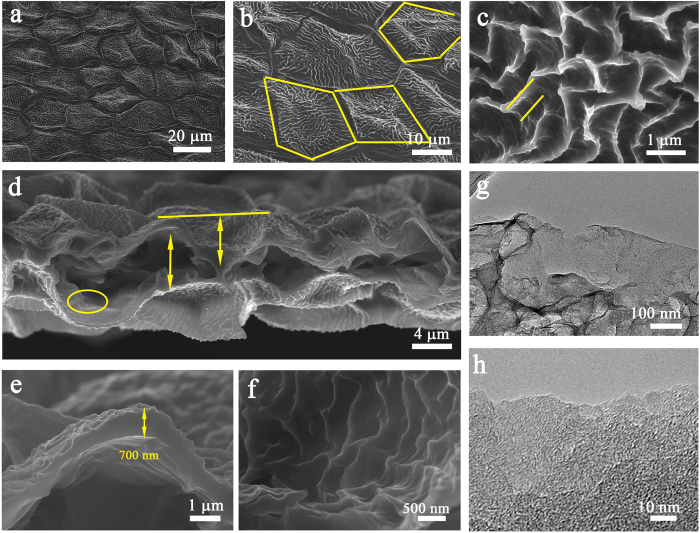
SEM and TEM images of AWCMs. (**a**) SEM and (**b**,**c**) magnified SEM images of the AWCM, showing the wrinkled surface. (**d**) SEM and (**e**,**f**) magnified SEM images of the cross-section of the AWCM. (**g**,**h**) TEM and magnified TEM images of the AWCM.

To reveal the structure of the carbonized membrane, scanning electron microscopy (SEM) and transmission electron microscopy (TEM) of the AWCM were performed. As shown in Fig. 2a,b and Figure S1, both AWCM and WCM inherited the hierarchy structure of the original petals. As indicated with yellow lines in Fig. 2b, the surface is made up of many wrinkled quadrangle, pentagonal and hexagonal domains of about 10 μm in width and 20 μm in length. From the magnified SEM image of a single domain in Fig. 2c, a lot of subwrinkles can be clearly observed. The height of the wrinkle is around 5–10 nm and the distance between each wrinkle is around 3–5 μm. The inner structure of the AWCM is displayed by the SEM image for the cross-section part from Fig. 2d to Fig. 2f From Fig. 2d, each carbon membrane is made up of two pieces of wrinkled film stacking together with an inner gap of 1–3 μm between the films. The thickness of a single wrinkled film is only 700 nm, and the total thickness of the membrane is 5–10 μm. This small thickness guaranteed the flexibility of the carbon membrane to be flexible electrode for EDLCs. From the magnified image indicated by the circle in Fig. 2d, one can see there are also numerous smaller wrinkles in the inner side of the single membrane, as shown in Fig. 2f. This hierarchical porous structure endows the AWCM an excellent electrode to store ions in supercapacitors. TEM image in Fig. 2g and h provide further insight for the microstructure of the AWCM, where a thin film with short-range aligned crystal lattice indicates an amorphous carbon structure^26^. Compared with the TEM image of the WCM (see Figure S2), more disordered carbon regions are observed, indicating that numerous pores are fabricated in the activation process, which reduced the ion-transport path and further increased the rate performance of the SCs^23^.

### Structures of wrinkled carbon membrane

**Figure 3:**
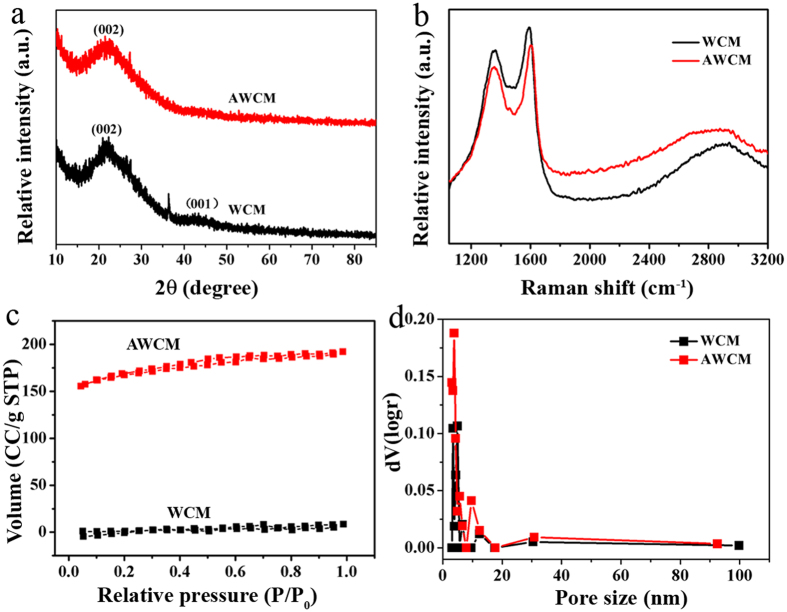
Structural analysis of the WCM and AWCM. (**a**) XRD patterns and (**b**) Raman spectrum comparison of the WCM and AWCM. (**c**,**d**) N_2_ adsorption and desorption isotherm and pore size distribution of the WCM and AWCM.

Further structural information was investigated and the results are shown in Fig. 3. Figure 3a presents the X-ray diffraction (XRD) patterns of the WCM and AWCM. Two weak and broad peaks at 22.2° and 44° demonstrate the diffractions peaks of (200) and (100) planes of graphite^18^. Compared with WCM, the diffraction peak at 22.2° of the AWCM is broadened, suggesting the lower graphitization degree of the AWCM^20^, which is in accordance with the result of TEM. The Raman spectroscopy in Fig. 3b further confirms the lower degree of graphitization after activation. The two peaks corresponding to D band and G band locate at 1358 and 1589 cm^−1^ for WCM and 1350 and 1609 cm^−1^ for ACWM. The I_D_/I_G_ increased from 0.79 to 0.88, indicating that the activation process has successfully increased the number of disordered carbon and lowered the graphitization degree^23^. For SCs, SSA and pore structure are two critical factors that decide their specific capacitance and rate performance^27^. In order to investigate the pore structure before and after activation, N_2_ gas adsorption-desorption measurement was performed and the corresponding isotherms are provided in Fig. 3c. In comparison with WCM, the N_2_ adsorption volume of AWCM increased greatly from 10 to 191.5 cubic centimeters (CCs) at one standard pressure, indicating a rapid increase in SSA. Calculated by the standard BET method, the SSA of the AWCM is 509 m^2^/g, which is 79 times higher than that of WCM (6.44 m^2^/g), confirming that simply heating in air can efficiently increase the SSA and maintain the mechanical strength of the petals at the same time. The pore size distribution in Fig. 3d shows that the intensity of peak for the pores at around 3.6 nm increased greatly for AWCM. The introduction of these pores facilitates the transport of the ions through the electrode materials and further increases their specific capacitances. In order to obtain the chemical composition of the WCM and AWCM, the samples were analyzed by X-ray photoelectron spectroscopy (XPS). As can be seen Figure S3, the content of the oxygen increased from 6.77% for WCM to 14.41% for AWCM by atoms. This increased oxygen content indicates that some carbon atoms are oxidized and many defects are created, which is in agreement with the result shown in Fig. 3b. The introduction of these oxygen containing functional groups is also beneficial to increase the specific capacitance^28^.

### Electrochemical evaluation of as-fabricated electrodes

**Figure 4:**
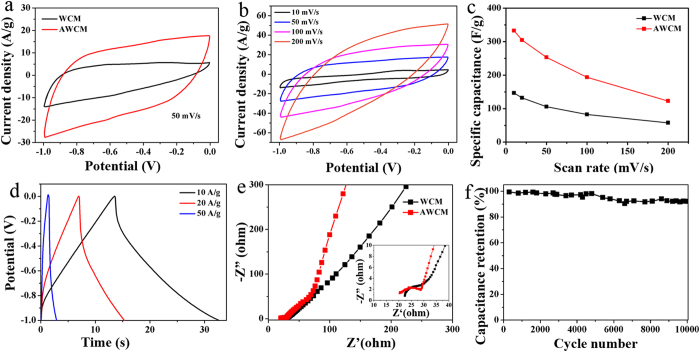
Electrochemical performance of the WCM and AWCM in EDLCs using aqueous electrolyte. (**a**) CV curves of the WCM and AWCM at a scan rate of 50 m V/s. (**b**) CV curves of the AWCM at different scan rates from 10 to 200 mV/s. (**c**) Specific capacitances calculated from CV curves as a function of scan rates. (**d**) GCD curves of AWCM at different discharge current densities. (**e**) Nyquist plots of the WCM and AWCM with the inset showing the high frequency region. (**f**) Capacitance retention of the AWCM over 10000 cycles.

The electrochemical performance of the WCM and AWCM as the electrode materials for EDLCs in a three-electrode system was evaluated in 0.5 M KCl electrolyte^29^,^30^,^31^. Figure 4a is the cyclic voltammogram (CV) curve comparison of the WCM and AWCM electrode at the same scan rate of 50 mV/s, where the area enclosed by the quasi-rectangular CV curve of the AWCM is much larger than that of the WCM. The calculated specific capacitances of the AWCM and WCM are 253 and 105 F/g, respectively, resulting from the increased interface between the carbon membrane and the electrolyte and improved ion acceleration. Figure 4b exhibits the CV curves of the AWCM electrode at the scan rate from 10 to 200 mV/s. It can be seen that AWCM shows better rectangular shape than WCM (see Figure S4), which results from the higher porosity of the AWCM. The calculated specific capacitances of the WCM and AWCM at different scan rates are provided in Fig. 4c, with the maximum value of 146.8 and 332.7 F/g at 10 mV/s. It can be observed that the specific capacitance of both WCM and AWCM decrease with the increase of the scan rate, which is related to the limited ion diffusion on the electrode surfaces due to the fast charging. However, the performance of the AWCM is always better than that of WCM. The rate performance can be further improved by optimizing the activation process to tune the pore structure of the AWCM. The galvanostatic charge-discharge (GCD) curves of the AWCM at varied current densities from 10 to 50 A/g are displayed in Fig. 4d. The lines are almost symmetrical with slight distortion, indicating its good capacitive behavior. Electrochemical impedance spectroscopy (EIS) was carried out to study the charge transfer resistance within the WCM and AWCM based electrode. The Nyquist plot was collected over the frequency range from 0.01 Hz to 100 kHz at an open circuit voltage. As can be seen in Fig. 4e, for the AWCM, the line at the low frequency is almost vertical while that for WCM is a slope, demonstrating its better ion diffusion into the electrode structure. Furthermore, the appearance of the semicircle at the high frequency in the inset image also indicates a porous structure of the AWCM and better electrolyte intrusion in the electrode, which further facilitates a faster ion transport in the AWCM than that of WCM. To investigate the stability of the electrochemical performance of AWCM, the cyclic stability measurement was carried out. As shown in Fig. 4f, AWCM exhibits an excellent cycling performance. At a charge-discharge current density of 20 A/g over 10000 cycles, the capacitance retention is as high as 92.3%. This excellent stability is attributed to the stable ion adsorption-desorption process at the interface between AWCM and electrolyte.

The temperature of pyrolysis and activation plays an important role for the electrochemical performance of the carbon membrane electrode. Different carbon membranes were prepared under different pyrolysis temperatures at 600, 800, 1000 and 1200 °C separately, and the electrochemical performance comparison is shown in Figure S5. One can see the electrochemical performance of the electrode decreases under a lower or higher temperature, indicating 1000 °C is an appropriate temperature for the pyrolysis of the petals. Besides, we also studied the influence of the activation temperature on the electrochemical performance of the electrode. As shown in Figure S6, when the activation temperature increased from 300 to 400 °C, the specific capacitance dropped from 253.01 to 166.46 F/g. Therefore, the best activation temperature for the carbon membrane electrode is 300 °C.

**Figure 5:**
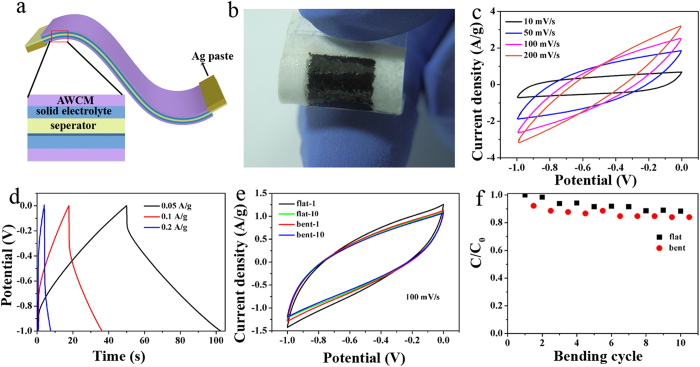
Electrochemical performance of the AWCM-based all-solid supercapacitor. (**a**) Schematic illustration of a flexible supercapacitor. (**b**) The image of the as-prepared supercapacitor. (**c**) CV curves of the device at different scan rates. (**d**) GCD curves of device obtained at different current densities. (**e**) CV curve comparison of the device at different bending times. (**f**) Capacitance retention of the device over 10 bending times.

To demonstrate the practical applications of AWCM, a flexible all-solid supercapacitor was fabricated, with AWCM as the electrode and H_3_PO_4_/Polyvinyl alcohol (PVA) gel as the solid electrolyte^32^,^33^,^34^. The schematic illustration of the flexible supercapacitor is demonstrated in Fig. 5a. Figure 5b is a picture of the as-prepared device, which is highly flexible and can be bent into different angles. The CV curves of the device at different scan rates from 10 to 200 mV/s are provided in Fig. 5c. A calculated specific capacitance of 154 F/g was obtained at a scan rate of 10 mV/s, which is higher than other porous carbon based supercapacitors^12^,^35^,^36^,^37^. Figure 5d presents the GCD curves of the device at different current densities from 0.05 to 0.2 A/g, where the symmetrical triangles indicate the good capacitive performance of the electrode materials in the device. To reveal the influence of the flexibility on the electrochemical performance of the device, a mechanical bending test of the device was performed. As can be seen in Fig. 5e, there is negligible change in the CV curves at a scan rate of 100 mV/s after 10 bending times, showing the stable electrochemical performance of the all-solid device towards the bending deformation. From the capacitance retention shown in Fig. 5f, it can be seen that the capacitance of the device after bending for 10 cycles is still more than 80% of the original one, which further confirms AWCM can work well as the electrode material for flexible EDLCs.

## Conclusions

In summary, we have prepared a freestanding AWCM from abundant and renewable flower petals by a thermal pyrolysis and further activation process. The AWCM maintains the shape and mechanical strength of the original petals and inherites their wrinkled microstructure on the surfaces. With a thickness of only 5–10 μm, the AWCM is flexible and can work as a binder and current collector-free electrode for SCs. With a three-electrode system in KCl electrolyte, the AWCM shows a specific capacitance of 332.7 F/g with an excellent cycling performance. The flexible all-solid supercapacitor with AWCM electrode shows a high specific capacitance of 154 F/g and good bending stability, where negligible capacitance decay was observed after 10 bending cycles. This provides a cost-effective and efficient electrode material for the energy storage for the flexible device.

## Methods

### Fabrication of WCM

Cherry blossom petals were collected on campus of Zhengzhou University and dried under a pressure between 200–500 Pa. The dried petals were put into a tube furnace and carbonized at 1000 °C for 1 h under Ar at a flow rate of 100 sccm. The temperature ramping rate of the furnace is 10 °C/min. To avoid curling, the petals were fastened between graphite slides during the carbonization process. After the reaction, the carbonized petals were immersed in 4.0 M HCl for 48 h to remove the inorganic impurities.

### Fabrication of AWCM

The clean WCM was put into the tube furnace and heated at 300 °C in air for 1 h with a ramping rate of 10 °C/min. To make the sample fully exposed to the air, WCM was heated in a quartz boat directly without graphite slides clapping.

### Materials morphology and structure characterization

The scanning electron microscopy (SEM) and transmission electron microscopy (TEM) were performed on Hitachi S-4800 and FEI Tecnai F20 instruments, respectively. X-ray diffraction (XRD) patterns were collected on PANalytical X’Pert Powder instrument with Cu Kαirradiation. Raman spectras were collected using a Renishaw inVia Raman microscope with a laser wavelength of 514.5 nm. The N_2_ gas adsorption was measured with a Micromeritics accelerated surface area porosimetry (NOVA 4200e, China) auto adsorption analyzer. The N_2_ adsorption isotherms were obtained at 77 K, and the specific surface area (SSA) was obtained by Brunauer–Emmett–Teller (BET) analyses of the adsorption isotherms. X-ray photoelectron spectroscopy (XPS) measurement was carried out on an ESCALAB250Xi apparatus at base pressure of 1 × 10^−9^ mbar with and X-ray source of Al Kα.

### Preparation of the solid electrolyte

H_3_PO_4_ and polyvinyl alcohol (PVA) were used as the solid electrolyte in the supercapacitor. At first, 2 g H_3_PO_4_ and 2 g PVA were put into 20 mL DI water in a round-bottom flask. Then the flask was put into a hot water bath of 90 °C under stirring for several hours until H_3_PO_4_ and PVA were completely dissolved and a clear solution was obtained.

### Fabrication of the supercapacitor

AWCM was first cut into rectangular strips of 6 mm*8 mm, then two strips of AWCMs were used as the electrodes for the supercapacitor. Ag wire was attached onto one side of each electrode to connect the AWCM to the external circuit, then gel PVA/H_3_PO_4_ was coated onto both sides of the AWCM electrodes until the AWCM are totally immersed into PVA/H_3_PO_4_ electrolyte. After that, the AWCM coated with PVA/H_3_PO_4_ was placed in air at room temperature for 12 h to evaporate the water in the electrolyte until a gel-like electrolyte was obtained. To separate the two electrodes, a piece of paper which is slightly larger than the electrode material was inserted between two pieces of AWCM. At last, the two pieces of electrodes were further fastened with insulate tape and a supercapacitor was obtained.

### Electrochemical measurements

Electrochemical measurements were carried out at room temperature using electrochemical workstation (CorrTest CS2350). Cyclic voltammetry and galvanostatic charge-discharge tests were performed in a voltage window of −1–0 V at different scan rates and current densities, respectively. The electrochemical impedance spectroscopy measurements were performed at frequency range from 100 kHz to 0.01 Hz. The mechanical flexibility test was carried out by manual control.

When tested in a three-electrode system, for a single electrode, its specific capacitance, Cs (F/g), can be calculated from the CV curves by


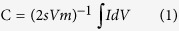


where *s* is the scan rate, *V* is the potential window, *m* is the mass of single electrode and *I* is current.

When asymmetric supercapacitor is charged, a voltage will build up across the two electrodes. The capacitance (C, F) of the device is calculated with the equation


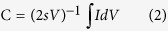


For an ideal symmetric supercapacitor, the specific capacitance, Cs (F/g) for the active material can be derived from the capacitance of the device


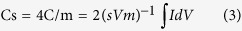


where *m* is the total mass of the active material (AWCM).

## Additional Information

**How to cite this article**: Yu, X. *et al*. Soft and wrinkled carbon membranes derived from petals for flexible supercapacitors. *Sci. Rep.* **7**, 45378; doi: 10.1038/srep45378 (2017).

**Publisher's note:** Springer Nature remains neutral with regard to jurisdictional claims in published maps and institutional affiliations.

## Supplementary Material

Supplementary Information
