# Corrigendum: Soft and wrinkled carbon membranes derived from petals for flexible supercapacitors

**DOI:** 10.1038/srep46932

**Published:** 2018-01-08

**Authors:** Xiuxiu Yu, Ying Wang, Li Li, Hongbian Li, Yuanyuan Shang

Scientific Reports 7: Article number: 4537810.1038/srep45378; published online: 03 31 2017; updated: 01 08 2018

In the original version of this Article, Li Li and Hongbian Li were incorrectly affiliated with ‘CAS Center for Excellence in Nanoscience, 11, Beiyitiao, Zhonguancun, Beijing, 100190, China’. The correct affiliations for Li Li and Hongbian Li are listed below.

CAS Center for Excellence in Nanoscience, National Center for Nanoscience and Technology, Beijing, 100190, China

University of Chinese Academy of Sciences, Beijing, 100049, China.

This error has now been corrected in the PDF and HTML versions of the Article.

