# Supplementary material for: Soft and wrinkled carbon membranes derived from petals for flexible supercapacitors
Source: Sci Rep. 2017 Mar 31;7:45378. doi: 10.1038/srep45378 (PMC5374445; doi:10.1038/srep45378)
Supplement: Supplementary Information [file srep45378-s1.doc]

**Supplementary information**

**Soft and wrinkled carbon membranes derived from petals for flexible supercapacitors**

Xiuxiu Yu,^a^ Ying Wang,^a^ Li Li,^b^Hongbian Li, *^b^ Yuanyuan Shang*^a^

^a^School of Physical Engineering, Zhengzhou University, Zhengzhou, Henan 450052, China

^b^National Center for Nanoscience and Technology, 11, Beiyitiao, Zhonguancun, Beijing, 100190,China

E-mail: [lihb@nanoctr.cn](mailto:lihb@nanoctr.cn); [yuanyuanshang@zzu.edu.cn](mailto:yuanyuanshang@zzu.edu.cn)

**1. Supplementary Figures**

Figure S1: SEM image of the WCM

Figure S2: TEM image of the WCM

Figure S3: XPS survey curves of the WCM and AWCM

Figure S4: CV curves of the WCM at the scan rate from 10 mV to 200 mV/s in the potential window between -1 to 0 V.

Figure S5: Electrochemical performance of the carbon membrane electrodes prepared at different pyrolysis temperatures.

Figure S6. Electrochemical performance of the carbon membrane electrodes prepared at different activation temperatures.

**
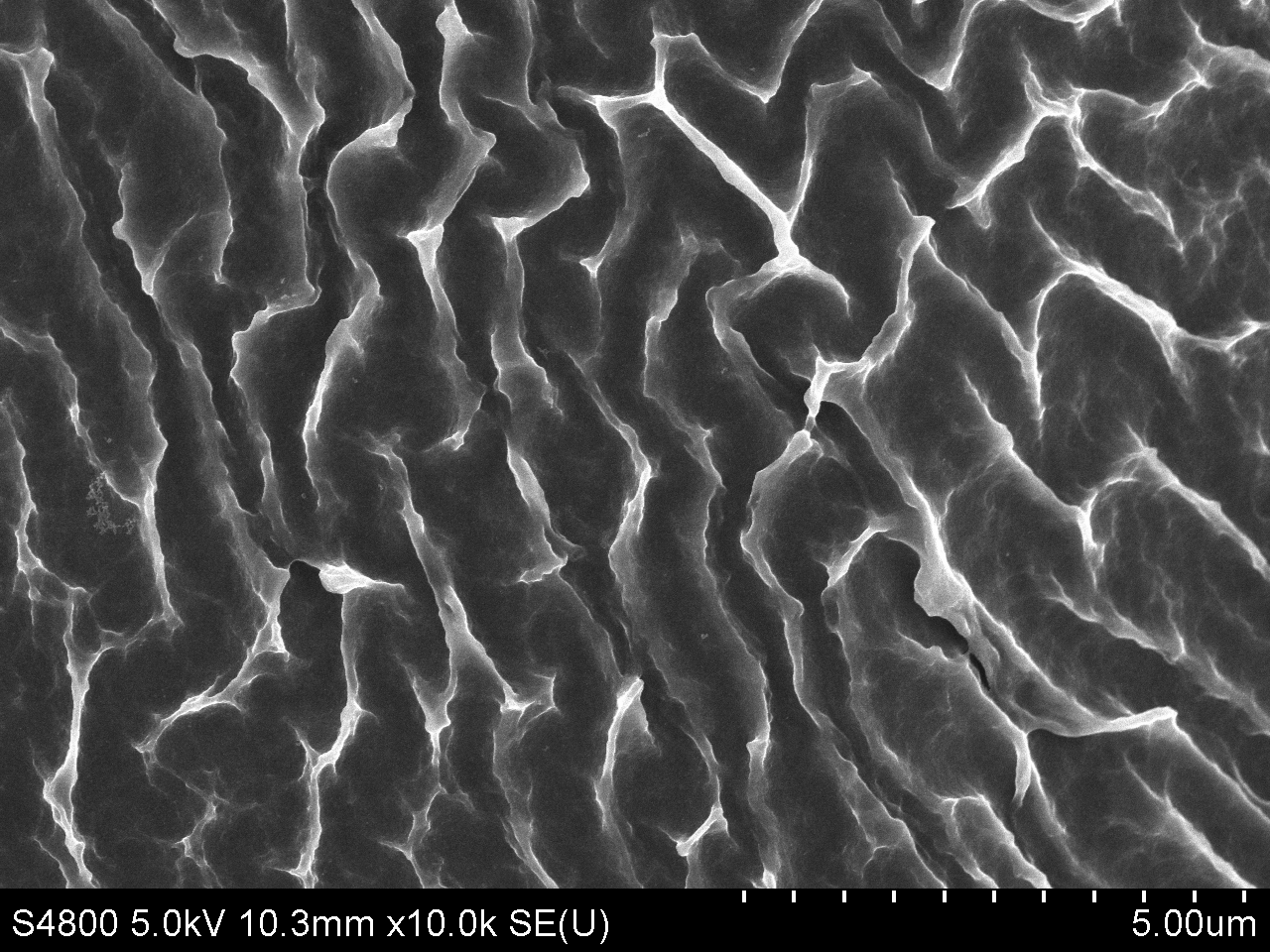
**

**Figure S1. Magnified SEM image of the WCM**

**
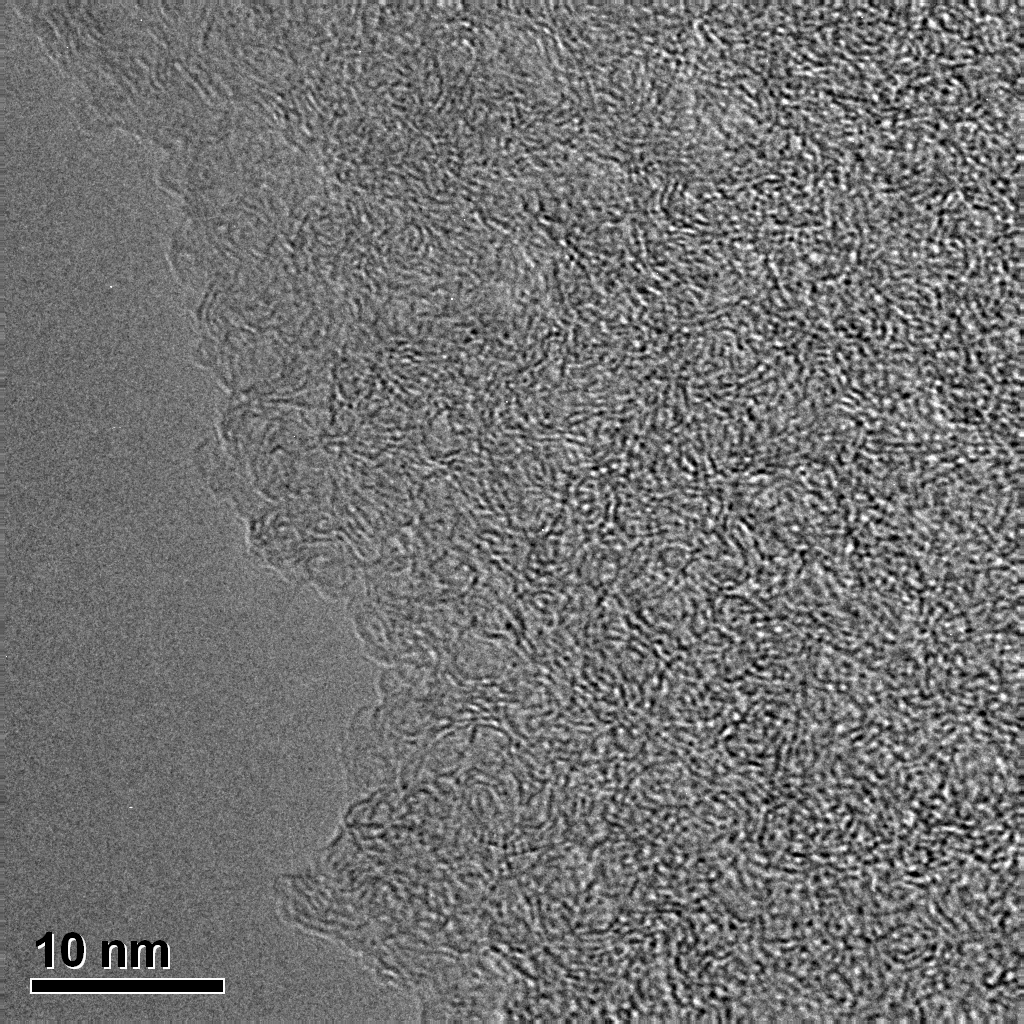
**

**Figure S2. Magnified SEM image of the WCM**

**
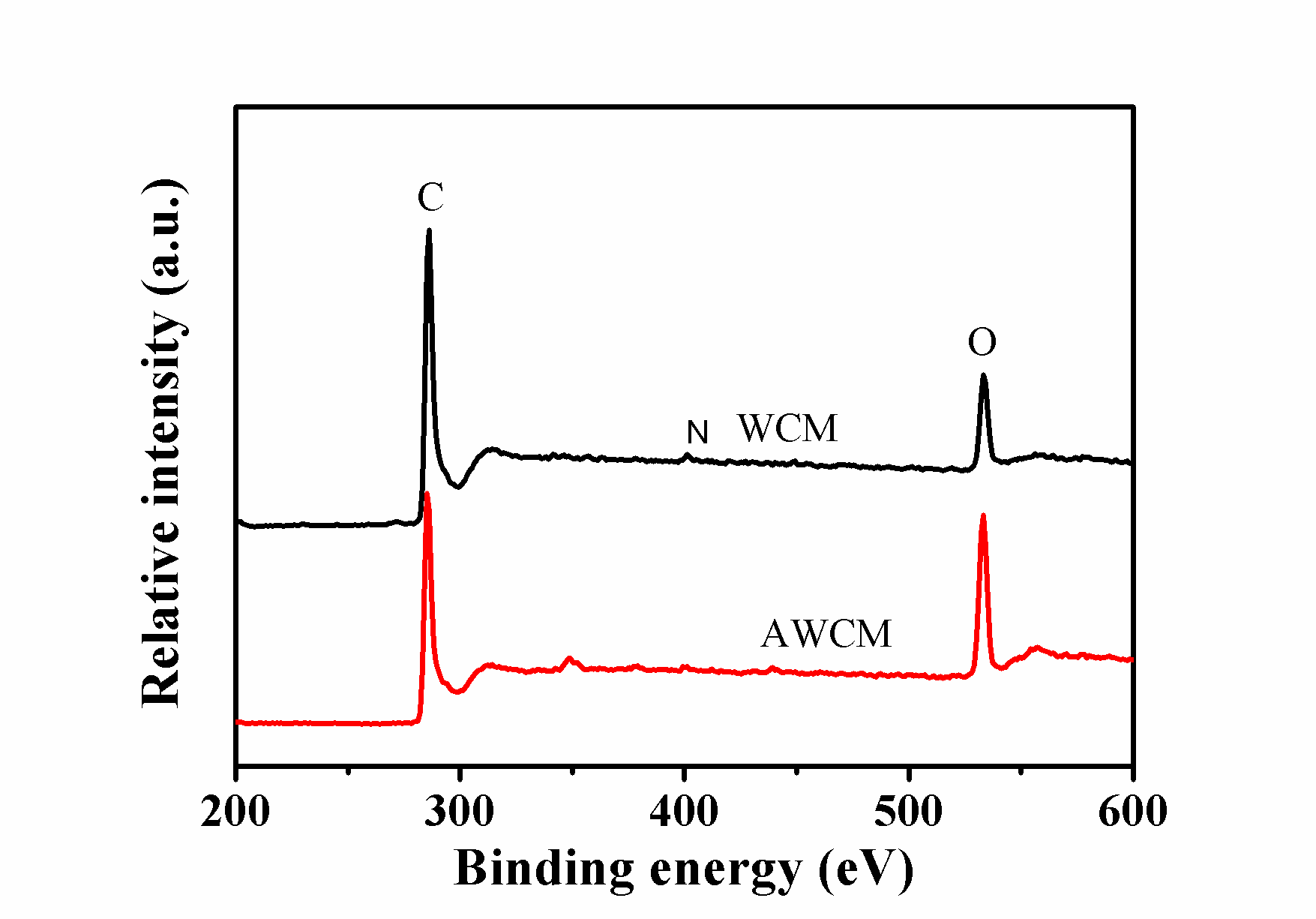
**

**Figure S3. XPS survey curves of the WCM and AWCM**

**
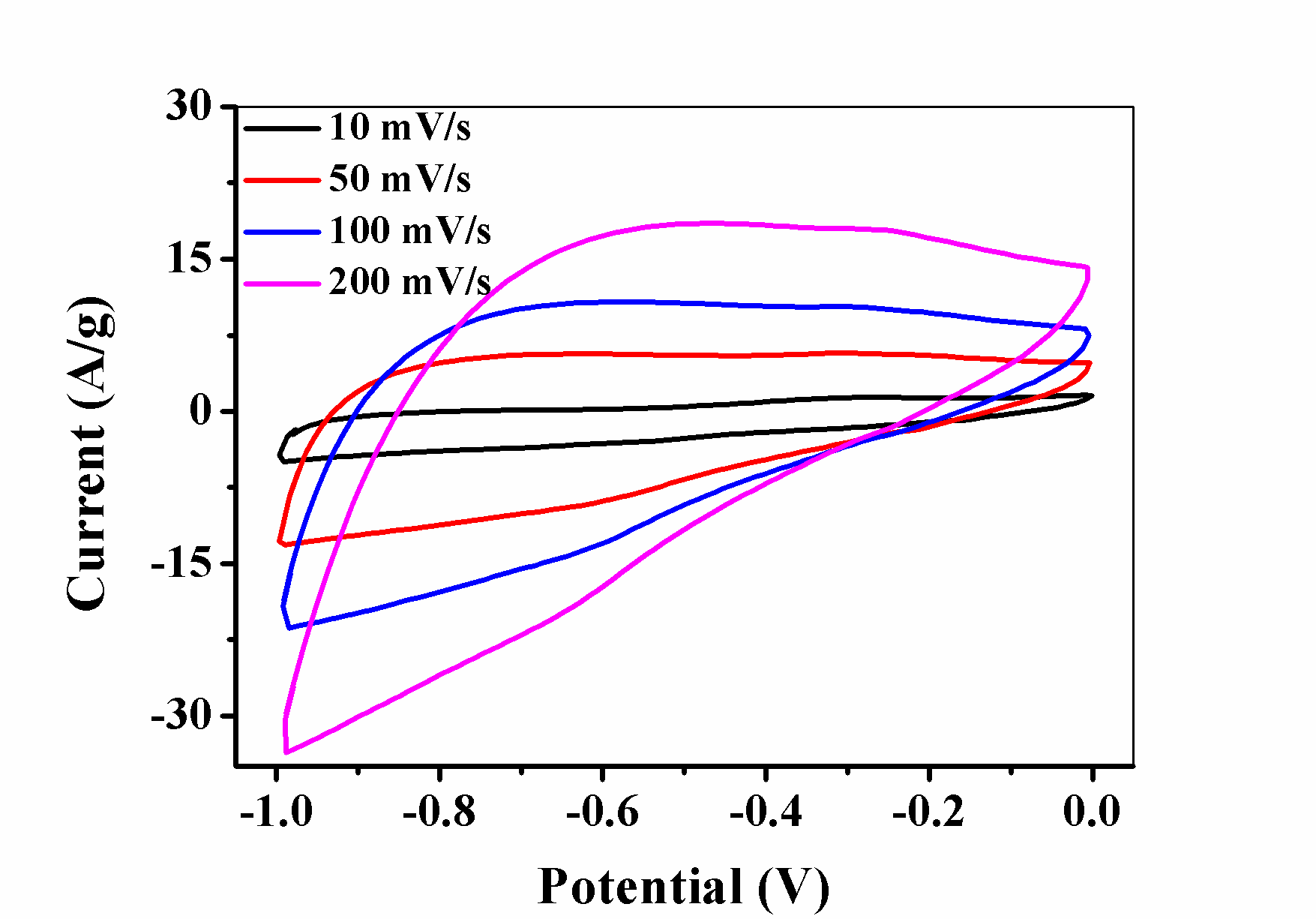
**

**Figure S4. CV curves of the WCM at the scan rate from 10 mV to 200 mV/sin the potential window between -1 to 0 V**


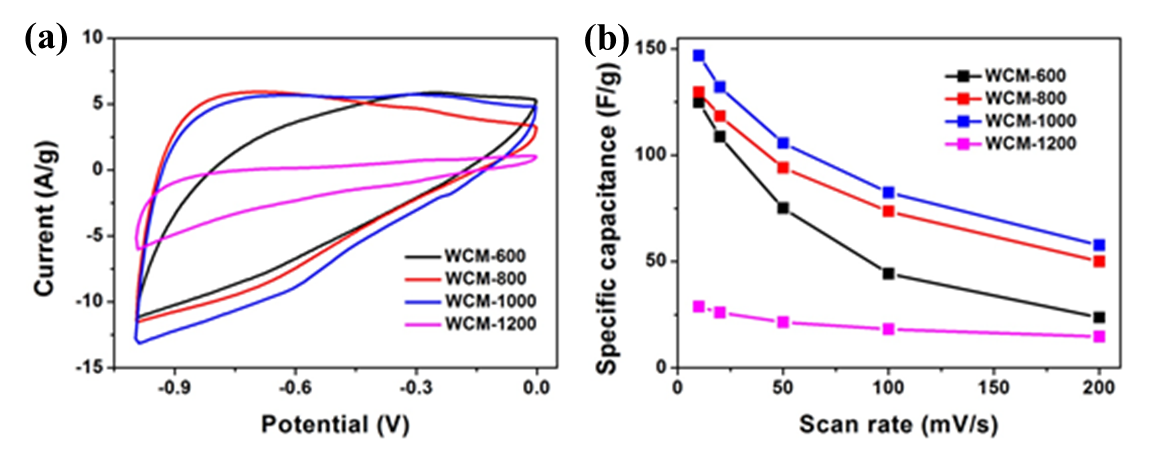


Figure S5. Electrochemical performance of the carbon membrane electrodes prepared at different pyrolysis temperatures. (a) Cyclic voltammogram curves of the WCMs obtained at different pyrolysis temperatures; (b) Specific capacitances of different WCMs as a function of scan rates.


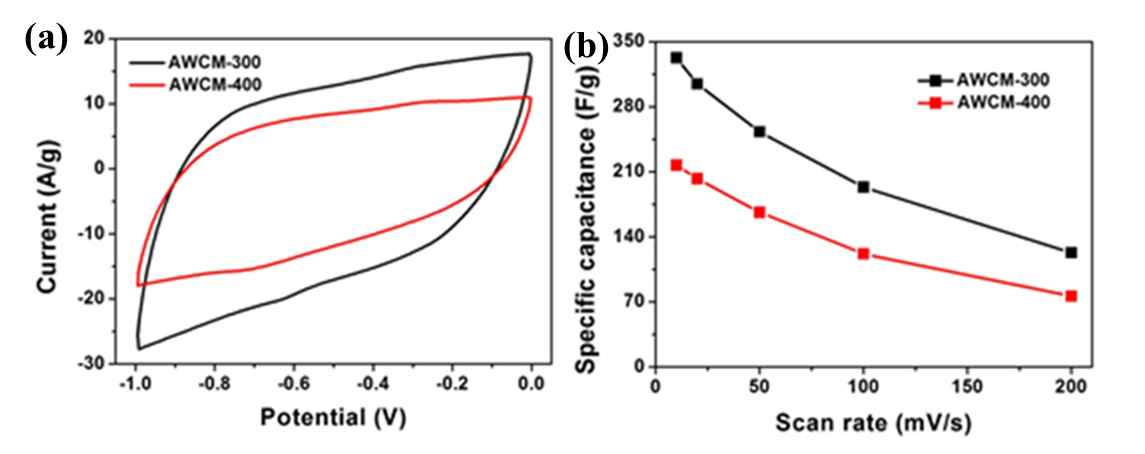


Figure S6. Electrochemical performance of the carbon membrane electrodes prepared at different activation temperatures. (a) Cyclic voltammogram curves of the AWCMs obtained at different activation temperatures; (b) Specific capacitances of different AWCMs as a function of scan rates.
